# Interplay between circadian clock and viral infection

**DOI:** 10.1007/s00109-017-1592-7

**Published:** 2017-09-30

**Authors:** Xiaodong Zhuang, Srinivasa Bhargav Rambhatla, Alvina G. Lai, Jane A. McKeating

**Affiliations:** 1 Nuffield Department of Medicine, University of Oxford, Oxford, UK; 2 Institute for Immunology and Immunotherapy, University of Birmingham, Birmingham, UK

**Keywords:** Circadian rhythms, Infectious disease, Viruses

## Abstract

The circadian clock underpins most physiological conditions and provides a temporal dimension to our understanding of body and tissue homeostasis. Disruptions of circadian rhythms have been associated with many diseases, including metabolic disorders and cancer. Recent literature highlights a role for the circadian clock to regulate innate and adaptive immune functions that may prime the host response to infectious organisms. Viruses are obligate parasites that rely on host cell synthesis machinery for their own replication, survival and dissemination. Here, we review key findings on how circadian rhythms impact viral infection and how viruses modulate molecular clocks to facilitate their own replication. This emerging area of viral-clock biology research provides a fertile ground for discovering novel anti-viral targets and optimizing immune-based therapies.

## Introduction

**Fig. 1:**
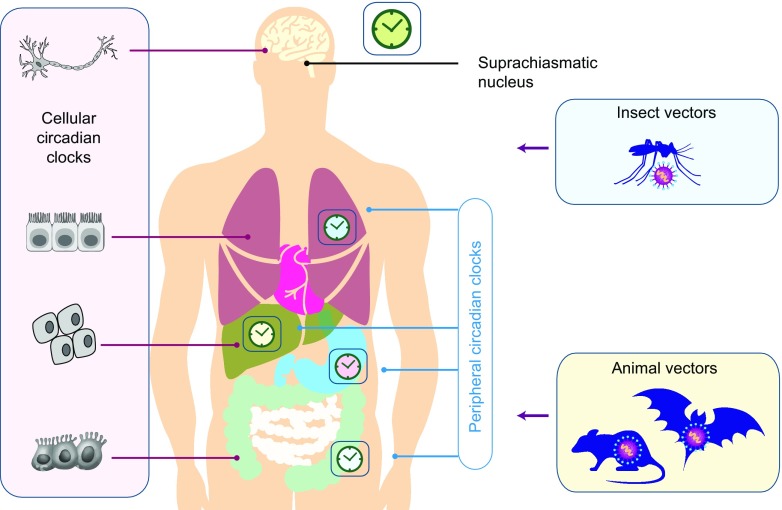
Central clock and peripheral clocks. The mammalian circadian clock consists of the central oscillator, located in the suprachiasmatic nucleus (SCN) of the hypothalamus, and peripheral clocks present in virtually all cells of the body. Light activates photoreceptors in the retina that are connected to the central SCN clock that synchronises and entrains peripheral circadian clocks via neural and endocrine pathways. The interplay between the circadian clocks of man and vectors that carry viral pathogens may impact on their capacity to transmit virus

**Fig. 2:**
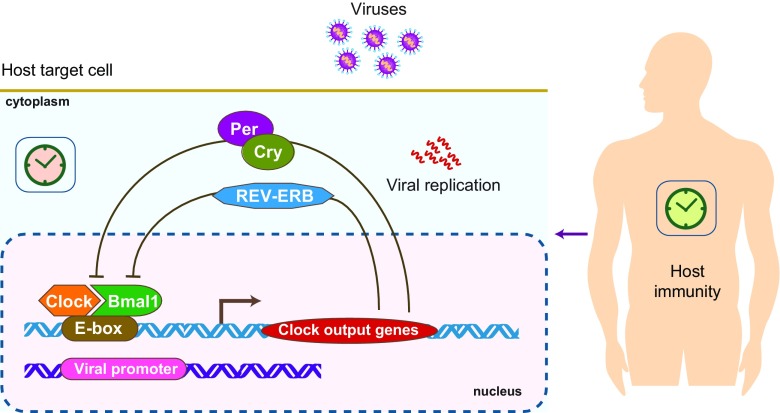
Molecular regulation of the circadian system. The master transcription factors CLOCK and BMAL1 form a heterodimer which binds the E-box in the promoter region and induce the expression of various gene products, including REV-ERB, Cryptochrome (CRY) and Period (PER) that supress BMAL1 and CLOCK expression, which constitute a negative feedback loop

Circadian rhythms are autonomous, self-sustaining, 24-h oscillations that synchronise physiological processes, such as sleep–wake cycles, hormone release, cell regeneration, fluctuations in body temperature and metabolism, to external environmental cues. These rhythmic processes are controlled by the circadian timekeeping system that consists of a central circadian clock located in the suprachiasmatic nucleus (SCN) that links to a network of peripheral clocks located in every tissue (Fig. 1). The mammalian clock circuitry receives entraining light signals from the retina that drive a transcriptional/translational feedback loop that is controlled by two activators (basic helix-loop-helix transcription factors CLOCK and BMAL1) and two repressors (Period (PER) and Cryptochrome (CRY)). CLOCK and BMAL1 form a heterodimer that binds E-box motifs in the promoter region of their target genes [1]. Gene products include several negative regulators that repress CLOCK/BMAL1 activity, which include PER1-3, CRY1-3 and REV-ERBα and β [2]. These negative feedback loops repeat approximately 24 h and form the basis of the rhythmic circadian regulatory pathways (Fig. 2). Microarray analyses show that 10% of genes in various tissues are regulated by the circadian clock [3]; furthermore, the identity of the oscillating transcripts differs between tissues. Recent studies highlight a role for clock transcription factors (TFs) to bind tissue-specific enhancers that are established early in development [4].

BMAL1 is a key component of the circadian clock [5]; mice deficient in BMAL1 show impaired circadian behaviour and many physiological abnormalities including glucose homeostasis, insulin production, accelerated aging (reviewed in [6]) and increased susceptibility to virus infections [7, 8]. Viruses are intracellular pathogens that require host cells to replicate and given the wide-ranging effects of these clock TFs on the cellular transcriptome, we would expect circadian clocks to play a role in defining host susceptibility to viral infections. However, our understanding of the molecular interplay between the circadian clock and viral replication is limited, and we have taken the opportunity to review the published literature in this area to highlight the need for further studies in this exciting area of viral-clock biology.

## The impact of the circadian clock on host anti-viral immunity

Many aspects of the innate and adaptive immune systems are under circadian control [9, 10]. To prevent the immune system from being in a state of constant overdrive, it has evolved to anticipate when pathogens are most likely encountered. This phenomenon was elegantly demonstrated in a vesicular stomatitis virus (VSV) murine encephalitis model where mice infected at the beginning of the rest phase (Zeitgeber Time 0, ZT0) showed poorer survival (95% mortality rate) compared to those challenged at the start of the active period (40% mortality rate) [11], suggesting a role of circadian/diurnal regulation of host anti-viral responses and associated pathologies. Infecting mice at ZT0 was associated with an increased number of inflammatory cells and expression of chemokine (C-C motif) ligand 2 (CCL2). Furthermore, inhibiting the circadian repressor REV-ERBα with a synthetic antagonist increased CCL2 expression and virus-associated mortality during the active period, demonstrating a positive role for the circadian clock to regulate anti-viral immunity [11]. A recent report demonstrated the circadian regulation of immune cell trafficking where lymphocytes entered lymph nodes at the onset of the night phase and egressed from the tissue during the day, providing an additional level for the circadian clock to regulate host immunity to infectious agents [12].

Inflammatory lung diseases frequently show time-of-day variation in their severity, and a recent study from Gibbs and colleagues [13] showed that anti-bacterial immune responses in the lung are under circadian control. The authors discovered that pulmonary epithelial cells secrete CXC-chemokine ligand 5 (CXCL5) in a BMAL1-dependent manner that regulates neutrophil recruitment to the lungs. Of note, the Cxcl5 promoter activity showed no evidence of intrinsic circadian regulation but was repressed in a rhythmic manner by glucocorticoids (GCs). GCs display a robust circadian rhythm and regulate gene expression by binding and activating the glucocorticoid receptor, a transcription factor with wide ranging activities. The authors conclude that clock-controlled glucocorticoid receptor recruitment to the Cxcl5 promoter in epithelial club cells may explain the anti-inflammatory activity of steroids in the inflamed lung. This study provides an explanation for why disease symptoms show time-of-day variation in patients with inflammatory lung conditions. A recent report from Ehlers and colleagues studied the role of the circadian clock in regulating viral-associated lung disease [14]. Deletion of bmal1-exacerbated acute viral bronchiolitis caused by Sendai virus (SeV) and influenza A virus in mice. Importantly, bmal1 KO mice developed more extensive asthma-like airway changes post-infection, including mucus production and increased airway resistance, supporting a role for bmal1 in regulating lung immunity to common viral triggers of asthma.

In addition to regulating host immunity to viral infections, the efficacy of viral vaccination has been reported to be under circadian control in rodents and humans. Silver et al. reported that expression of pattern recognition receptor Toll-like receptor 9 (TLR9) that recognises bacterial and viral DNA is circadian regulated [15]*.* To assess the functional consequences of this observation, the authors immunised mice with ovalbumin and TLR9 ligand (CpG ODNs) adjuvant at various times of day. Vaccination at the time of increased TLR9 expression (ZT19) resulted in a greater immune response to ovalbumin, and this phenotype was lost in PER2 deficient mice. These observations are consistent with emerging evidence showing a role for the circadian clock to regulate host defences against bacterial pathogens [16] and suggest that vaccine efficacy against DNA viruses can be enhanced by manipulating the time of day for vaccination.

Phillips et al. showed that patients immunised in the morning developed greater antibody responses to both hepatitis A and influenza vaccines [17]. More recently, Long et al. conducted a large randomised trial to examine the time of day influence on the magnitude of antibody response generated in older adults receiving their annual influenza vaccination. This study showed that morning vaccination significantly increased viral specific antibody responses compared with afternoon vaccination [18, 19], highlighting the need to consider the time of immunisations when designing vaccine efficacy trials. Modulating the circadian components or time of vaccination provides a simple approach to increase vaccine efficacy against a wide range of viruses.

## The effect of circadian regulators on viral infection

The liver is one of the most circadian-regulated organs with 20% of genes showing circadian patterns of expression [20]. Perturbing clock transcription factors alter hepatic metabolism and are associated with a variety of disorders including fatty liver disease, diabetes and hepatocellular carcinoma. Hepatitis B and C viruses infect the liver and are a leading cause of liver disease worldwide, and both viruses have been reported to exploit circadian clock-regulated pathways [21]. Benegiamo et al. showed that HCV infection decreased PER2 and CRY2 expression [22]. Importantly, overexpressing PER2 in permissive hepatocyte-derived cells reduced HCV RNA replication, and this associated with an increased expression of interferon-stimulated genes [22], suggesting that PER2 may regulate hepatocellular recognition of HCV-associated PAMPs and down-stream interferon expression. Of note, PER2 was reported to influence interferon gamma signalling [23, 24], a regulator of innate and adaptive immune responses against viral infection. Another potential step where clock-regulated pathways may impact HCV replication involves the circadian-regulated microRNAs. Gatfield et al. reported that a hepatic-specific microRNA, miR122, is negatively regulated by REV-ERBα [25] and given the essential role of miR122 in HCV replication [26, 27], it is plausible that HCV infection may be circadian regulated.

Two recent studies report increased replication of herpes, influenza [7], respiratory syncytial virus (RSV) and parainfluenza type 3 viruses [8] in *bmal1-/-* mice. Importantly, both studies confirmed an anti-viral role for BMAL1 using in vitro viral replication models that lack systemic circadian cues or host defenses, supporting a model where BMAL1 regulates cellular factors that are essential for viral replication. A comprehensive proteomic analysis of wild type and *bmal1*-/- primary cells revealed an enrichment of proteins involved in protein biosynthesis, endoplasmic reticulum function and intracellular vesicle trafficking [7]; all pathways are important for various steps in the viral life cycle. Collectively, these studies suggest that low-BMAL1 expression may promote viral replication. In addition to its daily oscillations, *Bmal1* shows seasonal variation in human blood samples with the lowest levels observed during the winter months [28] coinciding with the peak season for respiratory viral epidemics [29].

Herpes simplex virus (HSV) is a DNA virus whose gene expression is limited by histone deacetylation. CLOCK can function as a histone acetyltransferase, and Kalamkovi et al. reported that HSV hijacks this clock repressor to facilitate transcription of viral genes, where depletion or silencing CLOCK reduced viral protein expression [30, 31]. The authors demonstrate that CLOCK is an integral component of the viral transcriptional machinery due to its association with the transcriptional complex (ICP4, ICP27, ICP22 and TFIID). It is interesting to consider an earlier report showing that HSV-encoded viral transactivator ICP0 associates with BMAL1 [32], providing a mechanism for the virus to interact with CLOCK and to link viral transcription to cellular circadian time.

Glucocorticoids (GCs) regulate carbohydrate, lipid and protein metabolism and are widely used as anti-inflammatory agents. GCs are secreted from the adrenal gland in a rhythmic circadian fashion, and their peak expression coincides with the onset of the active phase, suggesting a role in synchronizing signals between the SCN and peripheral tissues. GCs regulate gene transcription by activating the glucocorticoid receptor that binds GC response elements (GRE) in target gene promoters [33]. Of note, GCs have been reported to increase HIV transcription by interacting with a GRE in the viral promoter [34], suggesting a mechanism for circadian regulation of viral replication. However, to date, no studies have shown a direct role for GCs to mediate circadian regulation of viral gene expression.

While individual molecular circadian components have been reported to modulate viral infection, melatonin, a potent regulator of the circadian rhythm secreted by the pineal gland [35] exhibits wide-ranging anti-viral activity [36]. Veneuzuelan equine encephalomyelitis (VEE) is a mosquito borne virus that is pathogenic in humans and horses. Melatonin administration confers protection in murine models of VEE, with decreased mortality and a delayed onset and time to death that associates with reduced virus in the periphery and brain. The authors reported increased expression of type II interferon post-melatonin administration and suggested that melatonin stimulates endogenous interferon expression [37]. RSV is a major respiratory pathogen in children that can result in severe lower respiratory tract infections. Huang et al. reported that oral administration of melatonin in a murine-RSV model can reverse virus associated pathological symptoms via inhibition of both oxidative stress and pro-inflammatory cytokine expression [38]. Clinical trials have shown that melatonin administration increased survival of newborn children from RSV-associated respiratory sepsis [39]. With the recent Ebola epidemic, melatonin was investigated as a potential treatment option, and Tan et al. reported that melatonin reduced many of the pathological changes associated with Ebola infection, such as endothelial disruption and disseminated intravascular coagulation [40]. There have been limited studies addressing the mechanism of melatonin anti-viral activity; however, a recent study from Kadena and colleagues [41] reported that melatonin down-regulated LPS induced expression of interferon regulatory factors and STAT signalling in macrophages. The lack of toxicity and ease of administration makes melatonin an attractive candidate for further study.

## Viral perturbation of the circadian clock

A growing awareness of ‘circadian disruption’-associated pathologies, such as a high risk of cancer in shift workers, highlights the potential for this pathway to be deregulated in viral-associated cancers [42–44]. Yang et al. reported that hepatitis B virus encoded X (HBx) perturbed several clock genes at the transcriptional level. Overexpression of HBx in the human HCC BEL-7404 cell line resulted in increased transcription of Clock, Per1 and Per2 and deceased Bmal1, Per3, Cry1-2 and cyclin-dependent Kinase Inhibitor ε (CKI ε) mRNA levels [45]. To validate these in vitro observations, the authors quantified transcript levels of selected clock genes in HBV-associated hepatocellular carcinoma tissue compared to matched peritumoral samples and noted significantly lower levels of Per1-3 and Cry2 mRNA, suggesting a role for HBV to perturb clock function. Recent studies confirm a dysregulation of circadian gene expression and associated signalling pathways in human hepatocellular carcinoma [46–49], highlighting a role for virus-induced dysregulation of the circadian pathway may contribute to cancer pathogenesis.

Human Immunodeficiency Virus (HIV) infection has been associated with the disruption of circadian-regulated physiological processes, including a blunting of systolic blood pressure decline from day to night [50]. Malone et al. reported that HIV-infected subjects show a progressive loss of CD4^+^ T-lymphocytes between the morning and evening coinciding with changes in the circadian pattern of serum cortisol levels [51]. To understand how HIV interacts with the circadian system, Duncan et al. studied the effect of the HIV encoded Transactivator of Transcription (Tat) protein on circadian activity. Using a doxycycline-induced Tat promoter in a murine system, the authors reported that chronic expression of Tat in the brain leads to a decreased amplitude of circadian rhythms in mice activity [52]. Furthermore, Tat expression induced phase shifts and reset the circadian rhythm in the night phase of mice. Finally, using whole-cell recordings of neurons in brain slices, Tat was shown to potentiate excitatory neurotransmission through NDMA receptor currents via enhanced glutamate transmission, providing a putative mechanism to modulate SCN entrainment to light [53, 54].

Studies with Simian Immunodeficiency Virus (SIV) reported perturbation of several circadian parameters of body temperature and activity parameters, where the amplitude of temperature and locomotor activity was significantly reduced [55]. The authors concluded that these signs were not due to the acute fever response induced by SIV infection, but rather due to chronic infection and speculated a role for PER1 whose mRNA levels were reduced in the SCN due to HIV/SIV-induced cytokine release [56, 57].

Other viral infections have been reported to influence circadian rhythms. Coxsackievirus A16 (CVA16) is one of the major causes of hand, foot and mouth disease, and transcriptomic analysis of infected human embryonic kidney cells identified differentially expressed miRNA target genes involved in circadian rhythm pathways, providing some insights into CAV16 induced pathogenesis [58]. Human T-Lymphotropic Virus Type-1 (HTLV-1) infection is associated with a reduced amplitude and 24 hour mean blood pressure and increase in 24 hour mean heart rate measurements [59]. Murine models of Influenza A infection report a reduced amplitude of circadian-regulated locomotor activity and an increase in circadian gene PER2 period that were associated with increased lung inflammation and injury [60].

Circadian rhythms are likely to impact viral transmission especially if one considers zoonoses where multiple species can act as viral reservoirs (Fig. 1). For pathogens such as Plasmodia that cause malaria, synchronizing their replication cycle with their host circadian rhythms contributes to their success [61]. The *Aedes aegypti* mosquito acts as a carrier for many viral infections such as dengue fever and Zika virus. Lima-Camara et al. reported that dengue virus infection increased the amplitude of rhythmic locomotor activity in the mosquito and speculated this would increase the vector’s capacity to transmit virus [62]. This circadian variation in activity has been reported for other vectors such as *Culicoides* biting midges that are vectors for African horse sickness and Bluetongue virus [63]. Considering the recent Zika virus epidemic in Brazil and the diurnal rhythm of the transmitting mosquito vector *A. aegypti*, circadian regulators may provide new therapeutic targets [64].

## Conclusions

Given the interplay between virus and the host circadian mechanism, host susceptibility to infection or disease is not only dependent on the infectivity of the viral inoculum, transmission route and length of exposure, but on the time of day when the pathogen is encountered. Understanding how viruses interact with host circadian rhythms has the potential to influence the treatment and clinical management of viral infections. In terms of circadian modulating therapies for treating or preventing viral infections, this could involve both pharmacotherapy and chronotherapy. Thus, it is possible that the efficacy of current anti-viral therapies could be improved by altering the time of drug administration. Recent advances in T cell engineering therapies show promising results using T cell receptor (TCR) or chimeric antigen receptor engineered T cells targeted against human viral antigens [65–67]. Interestingly, Fortier et al. showed that the engineered TCR activity displayed a circadian pattern upon antigen activation and this diurnal effect was blunted in CLOCK mutant mice [68], highlighting the importance of time of day when T cell therapies are administered to maximise anti-viral immunity.

How do viruses engage with the molecular clockwork and modulate timekeeping? The discovery that the transcriptional clock mechanism is universal and exists in essentially every cell of the body highlights the potential of these TFs to regulate host susceptibility to viral infection directly via binding viral DNA genomes or indirectly via controlling host gene expression (Fig. 2). The report from Collaco et al. showing that cytomegalovirus immediate-early promoter is circadian regulated in mice supports a model for clock TFs to directly regulate viral promoters [69]. At the simplest levels, the circadian activity of host metabolic and trafficking pathways can constrain viral replication. It will be interesting to ascertain whether the reported oscillations in viral replication are driven by clock-controlled changes in cellular metabolism—a neglected but growing area of interest in studies of viral-host interactions [70]. Viruses are well recognised to reprogram host cellular metabolism, and this has the potential to feedback and regulate core clock components. Studies showing that viruses can interact with core clock components provide a mechanism for viruses to exploit circadian variation. The clock machinery present within every cell offers a diversity of target pathways to modulate viral replication that appears conserved across diverse DNA and RNA viruses.
